# Indications and outcomes of glenoid osteotomy for posterior shoulder instability: a systematic review

**DOI:** 10.1177/17585732211056053

**Published:** 2021-12-02

**Authors:** Huda Sardar, Sandra Lee, Nolan S Horner, Latifah AlMana, Peter Lapner, Bashar Alolabi, Moin Khan

**Affiliations:** 1 Division of Orthopaedic Surgery, Department of Surgery, 3710 McMaster University, Hamilton, Ontario, Canada; 2 Division of Orthopaedic Surgery, Department of Surgery, 27337 The Ottawa Hospital, University of Ottawa, Ottawa, Ontario, Canada

**Keywords:** glenoid osteotomy, posterior shoulder instability, glenoid retroversion, shoulder instability, glenoid, shoulder

## Abstract

**Background:**

There is limited evidence examining glenoid osteotomy as a treatment for posterior shoulder instability.

**Methods:**

A search of Medline, Embase, PubMed and Cochrane Central Register of Controlled Trials was conducted from the date of origin to 28th November 2019. Nine out of 3,408 retrieved studies met the inclusion criteria and quality was assessed using the Methodological Index for Non-randomized Studies tool.

**Results:**

In 356 shoulders, the main indication for osteotomy was excessive glenoid retroversion (greater than or equal to approximately −10°). The mean preoperative glenoid version was −15° (range, −35° to −5°). Post-operatively, the mean glenoid version was −6° (range, −28° to 13°) and an average correction of 10° (range, −1° to 30°) was observed. Range of motion increased significantly in most studies and all standardized outcome scores (Rowe, Constant–Murley, Oxford instability, Japan Shoulder Society Shoulder Instability Scoring and mean shoulder value) improved significantly with high rates of patient satisfaction (85%). A high complication rate (34%, *n* = 120) was reported post-surgery, with frequent cases of persistent instability (20%, *n* = 68) and fractures (e.g., glenoid neck and acromion) (4%, *n* = 12). However, the revision rate was low (0.6%, *n* = 2).

**Conclusion:**

Glenoid osteotomy is an appropriate treatment for posterior shoulder instability secondary to excessive glenoid retroversion. However, the high rate of persistent instability should be considered when making treatment decisions.

**Level of Evidence:** Systematic review; Level 4

## Introduction

Shoulder instability is generally classified relative to direction (e.g. anterior, posterior or multidirectional) and duration (e.g. acute, chronic or recurrent).^1^ Posterior instability accounts for approximately 2–10% of shoulder instability cases and can manifest itself on a spectrum, ranging from mild subluxation to frank dislocation.^2–6^ Causation is often multifactorial including, glenoid retroversion, trauma, posterior labral tears, hyperlaxity and poor neuromuscular control.^7–9^ Due to the wide spectrum of clinical manifestations and aetiologies, it can be challenging to determine appropriate indications for treatment.^10^

Burkhead and Rockwood used a rehabilitative program as treatment for 140 unstable shoulders to demonstrate that non-operative treatment for posterior instability is successful in up to 89% of cases.^7^ However, certain risk factors such as increased glenoid retroversion predispose patients to failure of conservative treatment.^6,7^ For patients with posterior shoulder instability who have failed prior non-surgical treatments and soft tissue interventions, such as capsulolabral repairs, bony procedures become important. These procedures include posterior bone block or, in patients who have excessively retroverted glenoids, glenoid osteotomy (or glenoplasty).^2^ Glenoid osteotomy for posterior shoulder instability was first described by Scott in 1967.^11^ The procedure aims to correct the retroversion and achieve centric loading of the glenohumeral joint to re-center the humeral head in its appropriate location.^2,8,10,11^

There is limited evidence on the management of posterior shoulder instability.^3^ To our knowledge, this is the first systematic review analyzing the indications and outcomes of glenoid osteotomy used to treat this condition. Through our analysis, we aim to evaluate its clinical effectiveness while providing some direction on which patients would most benefit from the surgical procedure.

## Methods

This systematic review was conducted following the *Cochrane Handbook for Systematic Reviews of Interventions* and reported according to the PRISMA guidelines.^12,13^ The protocol was registered to PROSPERO (CRD42020161836) an international registry for systematic reviews.

### Search strategy

PubMed, MEDLINE, EMBASE and The Cochrane Central Register of Controlled Trials were searched from database inception to 28th November 2019. MeSH and EMTREE terms were used in various combinations and supplemented with free text to increase search sensitivity. A hand search of the reference lists of articles was also conducted to identify potentially eligible articles for inclusion (Supplemental Appendix 1–4).

### Eligibility

Inclusion and exclusion criteria were established a priori. The inclusion criteria consisted of primary studies on glenoid osteotomy to treat posterior shoulder instability published in a peer-reviewed journal. Only studies on humans reported in the English language were considered and any geographical location, age, gender, ethnicity and publication year were deemed appropriate. Studies with co-morbidities (e.g. brachial plexus palsy which leads to excessive glenoid retroversion) were not included. There were no restrictions related to the length of follow-up and concomitant procedures. Case reports, cadaveric or artificial bone studies, animal studies, unpublished abstracts, posters, opinions, systematic reviews, literature reviews, meta-analyses, protocols, guidelines, letters to editors and comments were excluded.

### Study selection

Two independent reviewers (HS and SL) screened titles and abstracts to identify potential studies for the full-text review. Any discrepancies were addressed through discussion. If a consensus was not reached, a third blinded reviewer (NH) was consulted. This process was repeated at the full-text review stage; however, this time a reason for exclusion was documented. Interrater agreement was calculated using Cohen k (kappa) for both Level 1 and Level 2 screening. Agreement was categorized *a priori* as the following: k = 0.21–0.40 as fair agreement, k = 0.41–0.60 for moderate agreement, k = 0.61–0.80 for substantial agreement and k = 0.81–1.0 for near-perfect agreement.

### Data abstraction

Data were abstracted by two independent reviewers (HS and SL) using a pre-defined data abstraction form. Discrepancies were resolved through discussion and any disagreements were resolved through the assistance of a third reviewer (NH). Authors were contacted for missing data. In cases where similar studies were conducted by the same group, the most recent study was included after email consultation with the relevant corresponding author. Abstracted data included study characteristics, indications for glenoid osteotomy and relevant clinical outcomes observed post-operatively.

The primary outcome assessed in this systematic review is the overall effect of glenoid osteotomy on posterior shoulder stability. Secondary outcomes include an examination of glenoid version (negative angles represent retroversion while positive angles are indicative of anteversion), range of motion and overall function, pain and discomfort, complications, instability scores and patient satisfaction.

### Risk of bias assessment

The quality and risk of bias of the included studies were performed in duplicate by two independent reviewers (HS and SL) using the Methodological Index for Non-randomized Studies (MINORS) tool.^12–14^ MINORS score uses an ideal total score of 16 for non-comparative studies and 24 for comparative studies, evaluating 8 or 12 domains of bias, respectively.^14^ Methodological quality was categorized a priori as very low-quality evidence (score 0–6), low quality of evidence (score 7–10), fair quality (score 11–15) and high quality (score >16) evidence for non-randomized studies.^14^

## Results

### Study selection

**Figure 1.**
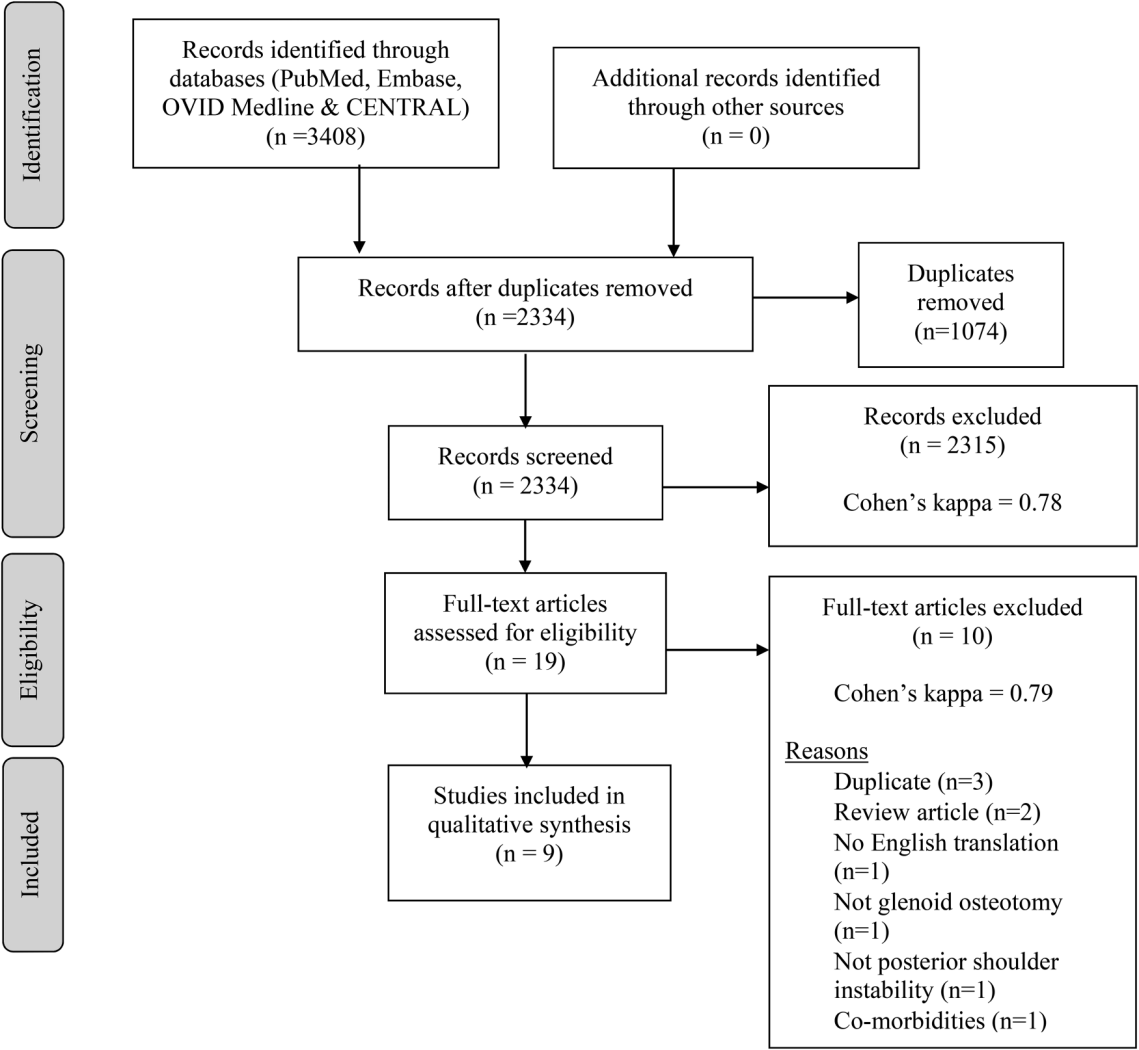
PRISMA diagram. Outline of selection and screening process based on the Preferred Reporting Items for Systematic Reviews and Meta-Analyses (PRISMA) guidelines.

The search strategy of the 4 databases retrieved 3,408 studies and 19 were identified to undergo full-text review. Nine studies met the inclusion criteria and were included in the final qualitative synthesis (Figure 1). A substantial agreement for both title and abstract screening (Cohen's kappa = 0.78) and full-text review (Cohen's kappa = 0.79) was observed.

### Study quality

The search resulted in nine studies with Level 4 evidence (Table 1), all of which adopted a case series design.^6,10,15–21^ The included non-comparative trials were of low methodological quality with a mean MINORS score of 6.5 (out of 16, SD 1.1). This excludes one study, which had a low MINORS score of 3 (out of 16) because researchers only had access to an abstract that lacked sufficient information about its methodology.^18^

**Table 1.** Risk of bias assessment of included studies.

			Evaluation of risk of bias												
			Methodological Index for Non-Randomized Studies (MINORS)												
Study	Levels of evidence	Type of study	Item 1	Item 2	Item 3	Item 4	Item 5	Item 6	Item 7	Item 8	Item 9	Item 10	Item 11	Item 12	TOTAL
Bessems and Vegter, 1995	4	Clinical non-controlled (case series)	2	0	0	1	0	1	2	0	NA	NA	NA	NA	6/16
Brewer et al., 1986	4	Clinical non-controlled (case series)	2	0	0	2	0	1	0	0	NA	NA	NA	NA	5/16
Graichen et al., 1999	4	Clinical non-controlled (case series)	2	2	0	1	0	1	0	0	NA	NA	NA	NA	6/12
Hawkins et al., 1996	4	Clinical non-controlled (case series)	2	1	0	1	1	1	1	0	NA	NA	NA	NA	7/16
Hawkins et al., 1984	4	Clinical non-controlled (case series)	2	0	0	1	1	1	1	0	NA	NA	NA	NA	6/16
Inui and Nobuhara, 2018	4	Clinical non-controlled (case series)	2	1	0	1	0	2	0	0	NA	NA	NA	NA	6/16
Lacheta et al., 2019	4	Clinical non-controlled (case series)	2	1	0	1	1	1	2	0	NA	NA	NA	NA	8/16
Ortmaier et al., 2017	4	Clinical non-controlled (case series)	2	0	0	1	1	2	2	0	NA	NA	NA	NA	8/16
			Only Abstract Accessible in English (Limited knowledge of study methodology)												
Pogorzelski et al., 2016	4	Clinical non-controlled (case series)	2	0	0	0	1	0	0	NA	NA	NA	NA	NA	3/16

NA: not applicable.

The items are scored 0 (not reported), 1 (reported but inadequate) or 2 (reported and adequate). The MINOR index evaluates different domains of bias using 8 (for non-controlled studies) and 12 (for controlled studies) categories, the global ideal score being 16 for non-comparative studies and 24 for controlled studies.

### Study characteristics

The studies included in this systematic review were conducted between 1984 and 2019, and about half of the studies (4/9) were published within five years of the search (Table 2).^6,15,16,18^ Of the nine included studies, two were conducted in Canada,^17,21^ three in Germany,^6,10,18^ while one was conducted in each of Japan,^15^ Austria,^16^ United States^19^ and the Netherlands.^20^

**Table 2.** Study characteristics.

Study	Country	Surgery details	Graft	Post-surgical treatment	Sample size (shoulders:patients)	Mean age (range)	Gender (Male:Female); % males	Mean follow-up (range)
Bessems and Vegter, 1995	The Netherlands	Posterior open wedge	Ilium	Thoracobrachial cast for 2–6 weeks (*n* = 6) Mitella for 2–4 weeks (*n* = 7) Physical therapy after immobilization for 10–26 weeks (*n* = 9)	13:10	19 years (15–27)	8:2; 80	9 years (1–16)
Brewer et al., 1986	United States	Posterior open wedge	Acromion (*n* = 4) Ilium (*n* = 1)	Sling & Codman exercises Active assisted exercises	5:5	16.2 years (11–20)	5:0; 100	
Graichen et al., 1999	Germany	Posterior open wedge	Ilium		32:32 But data on 16 patients reassessed post-op.			5 years (1.7–10.8)
Hawkins et al., 1996	Canada	Posterior open wedge	Ilium (*n* = 7) Acromion (*n* = 5)		12:12	23.8 years (21–35)	11:1, 91.6	5.1 years (2.6–8.3)
Hawkins et al., 1984	Canada	Posterior open wedge; capsular plication & infraspinatus tendon overlap		Spica cast for 4–6 weeks	17: not reported			
Inui and Nobuhara, 2018	Japan	Posterior open wedge	Ilium	Body spica for 2 weeks Isometric exercises Pillow to hold arm in slight abduction for 4–6 weeks Rotation exercises at 3 weeks Active resistance exercises from 6–8 weeks	249:211	20 years (13–47)	63:148; 29.9	7 years (5–21)
Lacheta et al., 2019	Germany	Posterior open wedge	Scapular spine	Immobilization & passive range of motion for 3 weeks Active-assisted motion from 3–6 weeks	12:11	25 years (17–40)		1.7 years (1.2–3)
Ortmaier et al., 2017	Austria	Posterior open wedge; performed more medially aiming at base of coracoid process.	Ilium	Sling for 4 weeks Finger and elbow exercises on first day Passive abduction & anterior flexion exercises started after 2 weeks Strengthening exercises after 6 weeks	10:8	41.5 years (24–51)	8:0; 100	2.8 years (2–4.3)
Pogorzelski et al., 2016	Germany	Posterior open wedge	Scapular spine or Ilium	Shoulder orthesis for 6 weeks Limited range of motion for 8 weeks. Over-head sports after 6 months.	6: not reported			

A total of 356 shoulders were included in this review. The mean of the average age of the included participants was 24 years old (range, 11–51). There were 13 shoulders in 10 patients examined in Bessems and Vegter,^20^ five shoulders in five patients in Brewer et al.,^19^ 12 shoulders in 12 patients in Hawkins et al.,^21^ 249 shoulders in 211 patients in Inui and Nobuhara,^15^ 12 shoulders in 11 patients in Lacheta et al.^6^ and 10 shoulders in 8 patients in Ortmaier et al.^16^ Graichen et al. reported data from 32 shoulders/patients, although only 16 were assessed postoperatively.^10^ In addition, Hawkins et al. and Pogorzelski et al. did not report the number of relevant patients; however, 17 and 6 shoulders were examined in these studies, respectively.^17,18^ The mean proportion of males was 80% based on five studies.^15,16,19–21^ Mean follow-up was between 1 and 10.8 years based on six studies (9 years [1–16] for Bessems and Vegter, 5 years [1.7–10.8] for Graichen et al., 5.1 years [2.6–8.3] for Hawkins et al., 7 years (5–21) for Inui and Nobuhara, 1.7 years [1.2–3] for Lacheta et al. and 2.8 years [2–4.3] for Ortamaeir et al.).^6,10,15,16,20,21^

All nine studies used the posterior open wedge technique. Seven studies used an autograft derived from the iliac crest,^10,15,16,18–21^ two from the acromion,^19,21^ two from the scapular spine,^6,18^ and one study did not report the autograft source.^17^ Furthermore, a variety of physiotherapy and shoulder braces (e.g. mitella and spicas) was used postoperatively for 2–26 weeks for most studies.^6,15–20^

### Indications for glenoid osteotomy

All studies consisted of patients presenting with recurrent posterior shoulder instability (Table 3). Four studies reported both unilateral (*n* = 44) and bilateral instability (*n* = 12).^10,17,19,20^ Excessive glenoid retroversion, defined as greater than or equal to approximately 10° based on Lacheta et al. and Pogorzelski et al., was reported in six studies,^6,10,16,18,19,21^ while one study also described their indication for glenoid osteotomy as ‘flatness of the glenoid’.^10^ Retroversion was determined using a variety of techniques, namely computed tomography, plain radiographs and magnetic resonance imaging.

**Table 3.** Indications for glenoid osteotomy^*^.

Study	Posterior instability	Mean glenoid retroversion (range)^**^	Dislocation or subluxation	History of trauma	Pain	Failed conservative treatment	Other
Bessems and Vegter, 1995	Unilateral (*n* = 1, 7.7%) Bilateral (*n* = 1, 7.7%)		Ligamentous laxity (*n* = 2, 15.4%)		Mild (*n* = 3, 23.1%)	Yes	Proximal humerus fracture 5 years prior to surgery (*n* = 1, 7.7%)
Brewer et al., 1986	Unilateral (*n* = 3, 60%) Bilateral (*n* = 2, 40%) Accentuated by increasing adduction and international rotation of arm	Glenoid angle: 73° (excessive: −10°)	Posterior displacement of humeral head by forward elevation of arm usually at 100°	No	No		Discomfort on elevation of arm, limited elevation of arm in forward flexion, inability to throw ball, swim and bench-press weights.
Graichen et al., 1999	Unilateral (*n* = 32, 100%)	−9.35° (−17° to −4.5°); *p* < 0.05 Flatness of glenoid	No capsular laxity or significant changes to labrum	No		Yes	Mild preoperative degenerative changes in glenohumeral joint (*n* = 1, 3.1%)
Hawkins et al., 1996	All	−8°	Posterior subluxation begins under voluntary control and later uncontrollable.	Yes (*n* = 5, 41.7%) No (*n* = 7, 58.3%)	Yes (*n* = 6, 50%)		Symptoms occur during forward flexion to horizontal, internal rotation and adduction
Hawkins et al., 1984	Unilateral (*n* = 8, 47.1%) Bilateral (*n* = 9, 52.9%)		Yes	Yes (*n* = undefined)	Yes (*n* = undefined)		
Inui and Nobuhara, 2018	*n* = 248, 99.9%		History/state of dislocation (*n* = 139, 55.8%)	No	Severe pain or pain at rest (*n* = 38, 15.3%); moderate pain during daily activities (*n* = 146, 58.6%); slight or occasional pain (*n* = 62, 24.9%); no pain (*n* = 3, 1.2%)		
Lacheta et al., 2019	All	−23.3° (−35° to −12°)	Posterior dislocations (*n* = 4, 33.3%) Chronic posterior subluxation (*n* = 3, 25%)				Early osteoarthritic changes (Samilson and Prieto stage ≥1): Stage 1 (*n* = 1, 8.3%); Stage 2 (*n* = 2), 16.6%; Stage 3 (*n* = 1, 8.3%)
Ortmaier et al., 2017	All	−16° (−31° to −11°)	Posterior static subluxation of the humeral head (≥5 mm)		Yes	Yes	Osteoarthritis (Samilson and Prieto stage ≥1): Stage 1 (*n* = 2, 20%); Stage 2 (*n* = 5, 50%); Stage 3 (*n* = 3, 30%)
Pogorzelski et al., 2016	All	−26.0° (−34.6°to −17.4°)		No		Yes	

^*^ *n* represents patients/shoulders.

Variables that do not have an indicated *n* value affected all patients.

% represents number of total cases.

^**^ negative angle implies retroversion while positive angle implies anteversion.

Two studies reported shoulder instability arising from trauma (*n* = 5, excluding one study)^17,21^ while five studies consisted of atraumatic shoulders (*n* = 299).^10,15,18,19,21^ Furthermore, four studies specified that patients had to have failed conservative treatment prior to surgical management.^10,16,18,19^

### Outcomes of glenoid osteotomy

#### Glenoid retroversion

The mean preoperative glenoid version was −15° (range, −35° to −5°) based on six studies.^6,10,16,18,19,21^ After surgery, an average glenoid version of −6° (range, −28° to 13°) was reported by five studies (Table 4).^6,10,16,18,21^ Mean version correction was observed to be 10° (range, −1° to 30°) as calculated by three studies.^15,16,21^ All three of these calculations are averages of reported means because raw data on each of the 356 shoulders is not available. A fourth study, Brewer et al., reported a range of correction values (21°–37°) and found that 60% of shoulders lost a mean correction in a version of 14°.^19^ This loss might be attributable to the screw fixation technique used in the study.^19^

**Table 4.** Outcomes of glenoid osteotomy^*^.

Study	Glenoid angle, version and correction	Dislocation, subluxation or recurring instability	Range of motion and overall function	Pain/discomfort	Osteoarthritis and complications	Instability scores	Satisfaction
Bessems and Vegter, 1995		Posterior subluxation towards right humeral head but stable after physical therapy (*n* = 1, 7.7%)	Normal (all) Slight limitation when throwing (*n* = 1, 7.7%) Return to work (all)	Mild during/after strenuous work (*n* = 3, 23.1%)	Slight degenerative changes (*n* = 1, 7.7%)	Rowe *(1981)* Excellent (*n* = 12, 92.3%) Good (*n* = 1, 7.7%)	
Brewer et al., 1986	*Glenoid angle*: 90.6 *Correction*: 21°–37° Some loss of correction (*n* = 3, 60%)	Grade III displacement of humeral head post-op but centralized by 1-year FU (*n* = 1, 20%) Stability after revision surgery (*n* = 1, 20%)	Normal (all) Excellent function	No discomfort			
Graichen et al., 1999	*Glenoid version*: −4.62° (−5° to 1°); *p* < 0.05	Recurrent instability with normal retroversion angle and glenoid depth (*n* = 2, 12.5%)	Limitation (*n* = 1, 6.3%) Play games that involved raising hands above head at a higher standard than before operation (*n* = 11, 68.8%) Carry out daily tasks satisfactorily (*n* = 14, 87.5%)		No loosening of implant, infection or nerve injury. Moderate degeneration (signs also seen prior to surgery (*n* = 4, 25%))	Good or excellent outcome either Constant–Murley or Rowe scores (*n* = 13, 81.3%)	Satisfied with the result (*n* = 12, 75%); marginally satisfied (*n* = 1, 6.3%); not satisfied (*n* = 3, 18.8%)
Hawkins et al., 1996	*Glenoid version*: 3° *Correction*: 10.8° (−1° to 24°)	Persistent instability (*n* = 2, 16.7%) Hill-Sach's lesion on humeral head (*n* = 1, 8.3%) Capsulorrhaphy in addition to osteotomy due to patulous posterior capsule (*n* = 2, 16.7%) Rounded/convex posterior portion of glenoid but no cartilage or labral damage (*n* = 1, 8.3%)	No significant change	Slight shoulder discomfort (*n* = 3, 25%)	Osteoarthritis (*n* = 1, 8.3%) Acromial fracture (*n* = 1, 8.3%) Intraarticular fracture (*n* = 1, 8.3%) Coracoid impingement (*n* = 1, 8.3%) Infected graft donor site (*n* = 1, 8.3%)		
Hawkins et al., 1984		Instability recurring on average 18 months post-op (*n* = 7, 41.2%)		Unremitting pain (cause undetermined, *n* = 1, 5.9%)	Avascular necrosis in the segment of glenoid requiring total shoulder arthroplasty. Involuntary subluxation (*n* = 1, 5.9%) External rotation contracture and osteoarthritis even after posterior release and anterior stabilization. Involuntary subluxation (*n* = 1, 5.9%) Ulnar nerve neurapraxia that resolved after 3 months (*n* = 1, 5.9%) Prolonged stiffness for 12 months (*n* = 1, 5.9%)		
Inui and Nobuhara, 2018	*Correction*: 9.5° (3°–12°); *n* = 60, 24.1%	Persistent instability (*n* = 44, 17.7%) Humeral head decentralized (*n* = 3, 1.2%) Improvement in joint congruency (*n* = 246, 98.8%) Additional stabilization for anterior instability (*n* = 12, 4.8%)	Decreased by mean of 4°–20° Statistically significant differences in all movements except external rotation Subacromial arthrolysis for restricted movement (*n* = 1, 0.40%)	Pre-op 5.6 (3.8, 0–20); *p* < 0.001 Post-op 13.1 (5.1, 0–20); *p* < 0.001	Osteoarthritic change due to previous surgery (*n* = 1, 0.40%) Intra-articular fractures (*n* = 7, 2.8%) Removal of body spicas due to numbness, paralysis of fingers or discomfort (*n* = 12, 4.8%) Disordered scapulohumeral rhythm (*n* = 8, 3.2%)	*Rowe* 36 points (0–60) to 88 points (40–100) *JSS-SIS* 47 points (12–67) to 81 points (14–100); *p*-value <0.001	Satisfied (*n* = 224), 90%; relatively satisfied (*n* = 6, 2.4%); dissatisfied (*n* = 19, 7.6%)
Lacheta et al., 2019	*Glenoid version*: −13° (−28° to −1°); *p* = 0.003	Recurrent instability (*n* = 1, 8.3%) No subluxations No revision surgeries			Asymptomatic glenoid neck fractures that did not affect scores (*n* = 3, 25%) Anterior cortical breach (*n* = 1, 8.3%)	*Rowe* 90 points (45–10) *Oxford instability* 44 points (21–48)	
Ortmaier et al., 2017	*Glenoid version*: −5° (−16° to 13°); *p* = 0.003 *Correction*: 10° (6°–30°).	Mean posterior static subluxation of humeral head from 5 mm (0–10 mm) preoperatively to 6 mm (range, 0–14 mm; *p* = 0.259) at final FU.	Abduction improved from 95° (45°–150°) to 113° (80°–180°; *p* = 0.004) Anterior flexion from 117° (50°–160°) to 143° (110°–180°; *p* = 0.006)		Osteoarthritis worsened from grade 2 to grade 3 (*n* = 2, 20%) Graft slipped out of osteotomy gap and stability achieved after graft re-inserted (*n* = 1, 10%)	*Constant*–*Murley* 45.1 points (24–71) to 64.1 points (44–92; *p* < 0.001). *Mean shoulder value* 60% (35%–95%).	Final outcome rated as very satisfying (*n* = 6, 60%), good (*n* = 2, 20%), and satisfying (*n* = 2, 20%)
Pogorzelski et al., 2016	*Glenoid version*: −11.2° (−20.6° to −2.2°)	Persistent posterior instability (*n* = 2, 33.3%) No revision surgeries					

^*^ *n* represents patients/shoulders; % represent the number of total cases; preoperation (pre-op), post-operation (post-op), follow-up (FU), Japan Shoulder Society Shoulder Instability Score (JSS-SIS).

^**^ negative angle implies retroversion while positive angle implies anteversion.

#### Range of motion and overall function

Two studies found that the range of motion returned to normal in all patients with excellent function (Table 4).^19,20^ Bessems and Vegter reported slight restrictions in a movement when throwing in a single patient (8%).^20^ Graichen et al. also had similar results reporting limitations in range of motion in 1 patient (6%).^10^ About 69% of shoulders (*n* = 11) were able to play games that involved raising hands above their heads at a higher standard than prior to the osteotomy and 88% of patients were able to carry out daily tasks satisfactorily.^10^

Shoulder range of motion also improved according to Ortmaier et al.;^16^ abduction improved from 95° (range, 45°–150°) to 113° (range, 80°–180°) (*p* = 0.004) and anterior flexion increased from 117° (range, 50°–160°) to 143° (range, 110°–180°) (*p* = 0.006).^16^ On the other hand, Hawkins et al. found no significant change in shoulder range of motion.^21^ Inui and Nobuhara found that range of motion decreased by a mean of 4°–20° and statistically significant differences were noted in all movements except external rotation.^15^

#### Satisfaction

The mean patient satisfaction rate was 85% based on three studies: 81% in Graichen et al., 92% in Inui and Nobuhara and 80% in Ortmaier et al. (Table 4).^10,15,16^

#### Instability scores

A variety of scores were reported in five studies (Table 4).^6,10,15,16,20^ The special dislocation (Rowe) score was reported as ‘excellent’ in 92% of cases (*n* = 12) and ‘good’ in 8% of cases (*n* = 1) in Bessems and Vegter.^20^ Inui and Nobuhara reported an increase in Rowe’s score from 36 points (range 0–60) to 88 points (range 40–100).^15^ A Rowe score of 90 points (range, 45–100) was found in Lacheta et al.^6^ The general shoulder function score (Constant–Murley) increased from 45 points (range, 24–71) to 64 points (range, 44–92) (*p* < 0.001) in Ortmaier et al.^16^ Graichen et al. reported a ‘good’ or ‘excellent’ outcome in either the Constant–Murley or the Rowe score in 81% of cases (*n* = 13).^10^ The Japan Shoulder Society Shoulder Instability Scoring (JSS-SIS) score increased from 47 points (range, 12–67) to 81 points (range, 14–100) (*p* < 0.001) in Inui and Nobuhara.^15^ The Oxford instability score was reported in Lacheta et al. as 44 points (range, 21–28) at follow-up.^6^ Ortmaier et al. reported a mean shoulder value of 60% (35%–95%).^16^ All these scores consistently show a positive impact of glenoid osteotomy on shoulder function. Minimal clinically important difference for these scores as they pertain to posterior shoulder instability cannot be determined due to the lack of literature on such findings.

#### Pain and discomfort

Five studies explicitly reported patients experiencing pain or discomfort (Table 4).^15,17,19–21^ Bessems and Vegter found the presence of mild pain during or after strenuous work in 23% of shoulders (*n* = 3).^20^ For Inui and Nobuhara, the preoperative and post-operative pain values as determined by the JSS-SIS system were 6 (SD 3.8, range 0–20) and 13 (SD 5.1, range 0–20) (*p* < 0.001, respectively), demonstrating an overall decrease in pain after surgery.^15^ No discomfort was observed in patients in Brewer et al.^19^ Hawkins et al. reported slight shoulder discomfort (*n* = 3),^21^ while Hawkins et al. reported unremitting pain of unknown cause (*n* = 1).^17^

#### Osteoarthritic changes

Degenerative changes were observed in six studies in 6% of shoulders at final follow-up (*n* = 18/317).^10,15,16,20,21^ However, prior osteoarthritic changes were reported in 40% of shoulders (*n* = 15/38) based on three studies, one of which solely focused on young patients with advanced osteoarthritis (Table 3 and Table 4).^6,10,16^

#### Complications

The overall complication rate was 34% (*n* = 120) yet revision surgery was reported in less than 1% of cases (0.6%, *n* = 2). About 20% of shoulders (*n* = 68/346) demonstrated recurrent posterior instability across eight studies.^6,10,15,17–21^ Recurring posterior instability observed in Graichen et al. was present even though the retroversion angle and glenoid depth were normal.^10^

The rate of fracture was 4% (*n* = 12/273) as reported by three studies.^6,15,21^ Intraoperative intra-articular fractures were observed in two studies – one case (8%) in Hawkins et al. and seven cases (3%) in Inui and Nobuhara.^15,21^ Intraoperative glenoid neck fractures were observed in 25% of cases (*n* = 3) in Lacheta et al., while an acromial fracture was observed in 8% of shoulders (*n* = 1) in Hawkins et al.^6,21^

Although Graichen et al. did not report any loosening of graft, infection or nerve injury, this was not the case in some other studies.^10^ Hawkins et al. had an infected donor graft side in 8% of cases (*n* = 1),^21^ Hawkins et al. had a case of avascular necrosis of the glenoid (6%) and another of ulnar nerve neurapraxia (6%),^17^ and Inui and Nobuhara required removal of body spicas in 5% of cases (*n* = 12) due to numbness, paralysis of fingers or discomfort.^15^ Approximately 3% of shoulders (*n* = 8) in Inui and Nobuhara also had a disordered scapulohumeral rhythm.^15^ Other relevant complications are outlined in Table 4.

## Discussion

This review identified mean glenoid version decreased with glenoid osteotomy with an average correction of 10°. Range of motion returned to normal in most patients and function significantly improved as measured by a variety of scoring systems (Constant–Murley, Rowe, Oxford instability, mean shoulder value and JSS-SIS). Common complications included persistent posterior instability in approximately 20% of patients and intraoperative iatrogenic fractures of the glenoid neck and/or acromion in 4% of cases.

Non-operative management of posterior shoulder instability is generally recommended as first-line therapy.^22,23^ Examples of conservative measures include immobilization, activity modification and physical therapy.^24–27^ In the medical literature, immobilization for 1–3 weeks has been shown to prevent recurring dislocation and joint instability while avoidance of activities requiring forward flexion, adduction and internal rotation has also been strongly encouraged.^25,26^ At the same time, strengthening exercises for the rotator cuff, scapula and posterior deltoid continue to provide therapeutic benefit.^27,28^ Although physical therapy improves disability in 70–80% of patients, surgery is generally ultimately recommended when symptoms of instability are persistent for longer than 3–6 months of rehabilitation or the patient has recurrent frank dislocations.^7,22,23,26,28–31^ In such a situation, posterior bone grafting, soft tissue repairs (i.e. post Bankart) and osseous procedures (i.e. glenoid neck posterior opening wedge osteotomy) can be performed.^29^ Recurring instability is observed in 6–10% undergoing soft tissue procedures and factors such as reverse Hill-Sachs lesions, glenoid bone loss and excessive glenoid retroversion contribute to this failure rate, making it necessary to perform a preoperative computed tomography (CT) scan to assess these clinical features.^32–39^

An analysis of glenoid version is key in posterior shoulder instability given that the posterior part of the labrum and the posterior side of the capsule are thinner and less developed as compared to their anterior counterparts.^10^ The average glenoid version analyzed by computed tomography scans of 410 normal shoulders from healthy volunteers is −1° ± 3° (range, −9° to 13°).^40^ This is relatively consistent with Graichen et al., which compared clinical data with 50 healthy controls and find a normal glenoid version of −4° (range −11° to 5°).^10^ Ultimately, an average post-operative version of −6° (range, −28° to 13°) found in this study suggests that glenoid osteotomies are effective at restoring glenoids to near normal version. It is also noteworthy that the instability observed in Graichen et al. was present even though the retroversion angle and glenoid depth were normal.^10^ This suggests that other causes of posterior shoulder instability must still be addressed when performing a glenoid osteotomy.

In this review, 40% shoulders (n = 15/38) had osteoarthritis prior to surgery^6,10,16^ and 6% of shoulders (*n* = 18/317) had degenerative changes post-operatively based on six studies.^10,15,16,20^ Osteoarthritic progressions were noted in some studies (e.g. Ortmaier et al.) and significant variability in the reported incidence of post-operative osteoarthritis was observed (e.g. 25% in Graichen et al. vs. 7.7% in Bessems and Vegter).^10,16,20^ Given these findings, we recommend a case-by-case review when deciding whether to perform glenoid osteotomy on patients with degenerative symptoms.^41^ Ideally, the procedure should be performed on patients with little to no osteoarthritis. Nevertheless, further analysis is needed to fully ascertain the impact of osteoarthritis on older patients undergoing glenoid osteotomy for posterior shoulder instability.

Overall, this review found that the range of motion returned to normal in most patients as they returned to their daily activities with a high degree of satisfaction and significantly improved instability scores. However, recurring instability was observed in 20% of shoulders (*n* = 68/346) possibly due to factors including humeral head or glenoid bone loss, convex glenoids and/or patient non-compliance.^6,10,15,17–21^

A variety of complications (34%) were also reported in studies included in this systematic review, including fractures of the glenoid neck and acromion, graft infection, avascular necrosis of the glenoid, numbness and finger paralysis.^6,11,16,20^ Fortunately, the revision rate was low (0.6%). This revision rate does not consider certain additional procedures performed due to complications, such as the removal of body spicas in Inui and Nobuhara.^15^

Ultimately, to reduce the high risk of complication, it is recommended that glenoid osteotomy be performed by experienced shoulder surgeons only.^6^ It is also suggested that the iliac crest (as opposed to the acromial bone) be used for the graft due to its higher density, secure fixation and easier preparation to match the desired size.^19^ The majority of studies in this review did not compare graft type to clinical outcomes comprehensively, and more clinical data are needed to reach a definitive conclusion.

This review does have limitations. Available evidence is of low quality and only one of the included studies was comparative in nature. Five of the nine studies were over 20 years old (1984–1999), and surgical techniques may have evolved.^10,17,19–21^ In addition, scores like Constant–Murley have poor discriminative properties for instability.^42^ Some studies did not conduct (or report) CT plane reformatting when measuring retroversion. Differences in angulation of the CT gantry make these measurements variable and prone to error.

Future studies should examine the impact of graft type and osteoarthritis on clinical outcomes. They should also use better tools for functional assessment and provide quantitative reports of each instability score. In addition, the need for controlled studies and higher level evidence is critical. Such analyses will provide research scientists and clinicians with a more complete view of the benefits and drawbacks of glenoid osteotomy. An understanding of the impact of mild glenoid retroversion on outcomes of soft tissue procedures (e.g. open capsular shift) would also be helpful. Lastly, the studies on other options for surgical management of recurrent posterior shoulder instability are required to discern the role of glenoid osteotomy in this armamentarium.

## Conclusion

Glenoid osteotomy for posterior shoulder instability is performed when excessive retroversion (<−10°) is present and conservative treatment has failed. Post-surgery range of motion generally returns to normal, and function improves consistently. However, the procedure is associated with significant complications including persistent instability and intraoperative fracture.

## Supplemental Material

sj-docx-1-sel-10.1177_17585732211056053 - Supplemental material for Indications and outcomes of glenoid osteotomy for posterior shoulder instability: a systematic review Click here for additional data file. Supplemental material, sj-docx-1-sel-10.1177_17585732211056053 for Indications and outcomes of glenoid osteotomy for posterior shoulder instability: a systematic review by Huda Sardar, Sandra Lee, Nolan S. Horner, Latifah AlMana, Peter Lapner, Bashar Alolabi and Moin Khan in Shoulder & Elbow

sj-docx-2-sel-10.1177_17585732211056053 - Supplemental material for Indications and outcomes of glenoid osteotomy for posterior shoulder instability: a systematic review Click here for additional data file. Supplemental material, sj-docx-2-sel-10.1177_17585732211056053 for Indications and outcomes of glenoid osteotomy for posterior shoulder instability: a systematic review by Huda Sardar, Sandra Lee, Nolan S. Horner, Latifah AlMana, Peter Lapner, Bashar Alolabi and Moin Khan in Shoulder & Elbow

sj-docx-3-sel-10.1177_17585732211056053 - Supplemental material for Indications and outcomes of glenoid osteotomy for posterior shoulder instability: a systematic review Click here for additional data file. Supplemental material, sj-docx-3-sel-10.1177_17585732211056053 for Indications and outcomes of glenoid osteotomy for posterior shoulder instability: a systematic review by Huda Sardar, Sandra Lee, Nolan S. Horner, Latifah AlMana, Peter Lapner, Bashar Alolabi and Moin Khan in Shoulder & Elbow

sj-docx-4-sel-10.1177_17585732211056053 - Supplemental material for Indications and outcomes of glenoid osteotomy for posterior shoulder instability: a systematic review Click here for additional data file. Supplemental material, sj-docx-4-sel-10.1177_17585732211056053 for Indications and outcomes of glenoid osteotomy for posterior shoulder instability: a systematic review by Huda Sardar, Sandra Lee, Nolan S. Horner, Latifah AlMana, Peter Lapner, Bashar Alolabi and Moin Khan in Shoulder & Elbow
