# Supplementary material for: Indications and outcomes of glenoid osteotomy for posterior shoulder instability: a systematic review
Source: Shoulder Elbow. 2021 Dec 2;15(2):117–31. doi: 10.1177/17585732211056053 (PMC10078812; doi:10.1177/17585732211056053)
Supplement: sj-docx-2-sel-10.1177_17585732211056053 - Supplemental material for Indications and outcomes of glenoid osteotomy for posterior shoulder instability: a systematic review [file sj-docx-2-sel-10.1177_17585732211056053.docx]

**Appendix 2. Search Strategy - Embase 1974 to 2019 search strategy**

<<Search ran on November 28, 2019>>

n = 1042

1. exp Shoulder/
2. exp Shoulder Fractures/
3. exp Shoulder Injuries/
4. exp Shoulder Joint/
5. exp Shoulder Dislocation/
6. shoulder.mp.
7. exp Glenoid Cavity/
8. exp Scapula
9. exp Joint Instability
10. glenoid.mp.
11. exp Arthroplasty, Replacement, Shoulder/
12. (surgery$ or surgeries or surgical or operat$).mp.
13. exp surgical Procedures, Operative/
14. exp. Osteotomy/
15. osteotom$.mp.
16. 1 or 2 or 3 or 4 or 5 or 6 or 7 or 8 or 9 or 10
17. 11 or 12 or 13
18. 14 or 15
19. 17 and 18
20. 16 and 19
21. limit 20 to (english and humans)
22. limit 21 to review
23. 21 not 22
