# Supplementary material for: Indications and outcomes of glenoid osteotomy for posterior shoulder instability: a systematic review
Source: Shoulder Elbow. 2021 Dec 2;15(2):117–31. doi: 10.1177/17585732211056053 (PMC10078812; doi:10.1177/17585732211056053)
Supplement: sj-docx-3-sel-10.1177_17585732211056053 - Supplemental material for Indications and outcomes of glenoid osteotomy for posterior shoulder instability: a systematic review [file sj-docx-3-sel-10.1177_17585732211056053.docx]

**Appendix 3. Search Strategy - PubMed search strategy**

<<Search ran on November 28, 2019>>

n = 581

((((((((("shoulder"[MeSH Terms] OR "shoulder fractures"[MeSH Terms]) OR "shoulder dislocation"[MeSH Terms]) OR "shoulder joint"[MeSH Terms]) OR "shoulder joint"[MeSH Terms]) OR "glenoid cavity"[MeSH Terms]) OR "glenoid cavity"[MeSH Terms]) OR "scapula"[MeSH Terms]) OR "joint instability"[MeSH Terms]) AND "osteotomy"[MeSH Major Topic]) AND (("arthroplasty, replacement, shoulder"[MeSH Terms] OR ((("surgery"[Subheading] OR "surgery"[All Fields] OR "surgical procedures, operative"[MeSH Terms] OR ("surgical"[All Fields] AND "procedures"[All Fields] AND "operative"[All Fields]) OR "operative surgical procedures"[All Fields] OR "surgery"[All Fields] OR "general surgery"[MeSH Terms] OR ("general"[All Fields] AND "surgery"[All Fields]) OR "general surgery"[All Fields]) OR ("surgical procedures, operative"[MeSH Terms] OR ("surgical"[All Fields] AND "procedures"[All Fields] AND "operative"[All Fields]) OR "operative surgical procedures"[All Fields] OR "surgeries"[All Fields]) OR ("surgical procedures, operative"[MeSH Terms] OR ("surgical"[All Fields] AND "procedures"[All Fields] AND "operative"[All Fields]) OR "operative surgical procedures"[All Fields] OR "surgical"[All Fields]) OR operat$[All Fields]) AND (("medical subject headings"[MeSH Terms] OR ("medical"[All Fields] AND "subject"[All Fields] AND "headings"[All Fields]) OR "medical subject headings"[All Fields] OR "mesh"[All Fields]) AND Terms[All Fields]))) OR "surgical procedures, operative"[MeSH Terms])
