# Supplementary material for: Indications and outcomes of glenoid osteotomy for posterior shoulder instability: a systematic review
Source: Shoulder Elbow. 2021 Dec 2;15(2):117–31. doi: 10.1177/17585732211056053 (PMC10078812; doi:10.1177/17585732211056053)
Supplement: sj-docx-4-sel-10.1177_17585732211056053 - Supplemental material for Indications and outcomes of glenoid osteotomy for posterior shoulder instability: a systematic review [file sj-docx-4-sel-10.1177_17585732211056053.docx]

**Appendix 4. Search Strategy - Cochrane Controlled Register of Trials (CENTRAL) search strategy**

<<Search ran on November 28, 2019>>

n = 1674

1. [mh "shoulder"]
2. [mh "shoulder fracture"]
3. [mh "shoulder injury"]
4. [mh "shoulder joint"]
5. [mh "shoulder dislocation"]
6. [mh "glenoid cavity"]
7. [mh "scapula"]
8. [mh "joint instability"]
9. (glenoid); ti, ab; kw
10. [mh "Osteotomy"]
11. (osteotom$); ti, ab; kw
12. {OR #1-#9}
13. {OR #10-#11}
14. #12 AND #13
